# Supplementary figures and images for: Equity in wastewater monitoring: Differences in the demographics and social vulnerability of sewered and unsewered populations across North Carolina
Source: PLoS One. 2024 Oct 10;19(10):e0311516. doi: 10.1371/journal.pone.0311516 (PMC11466389; doi:10.1371/journal.pone.0311516)

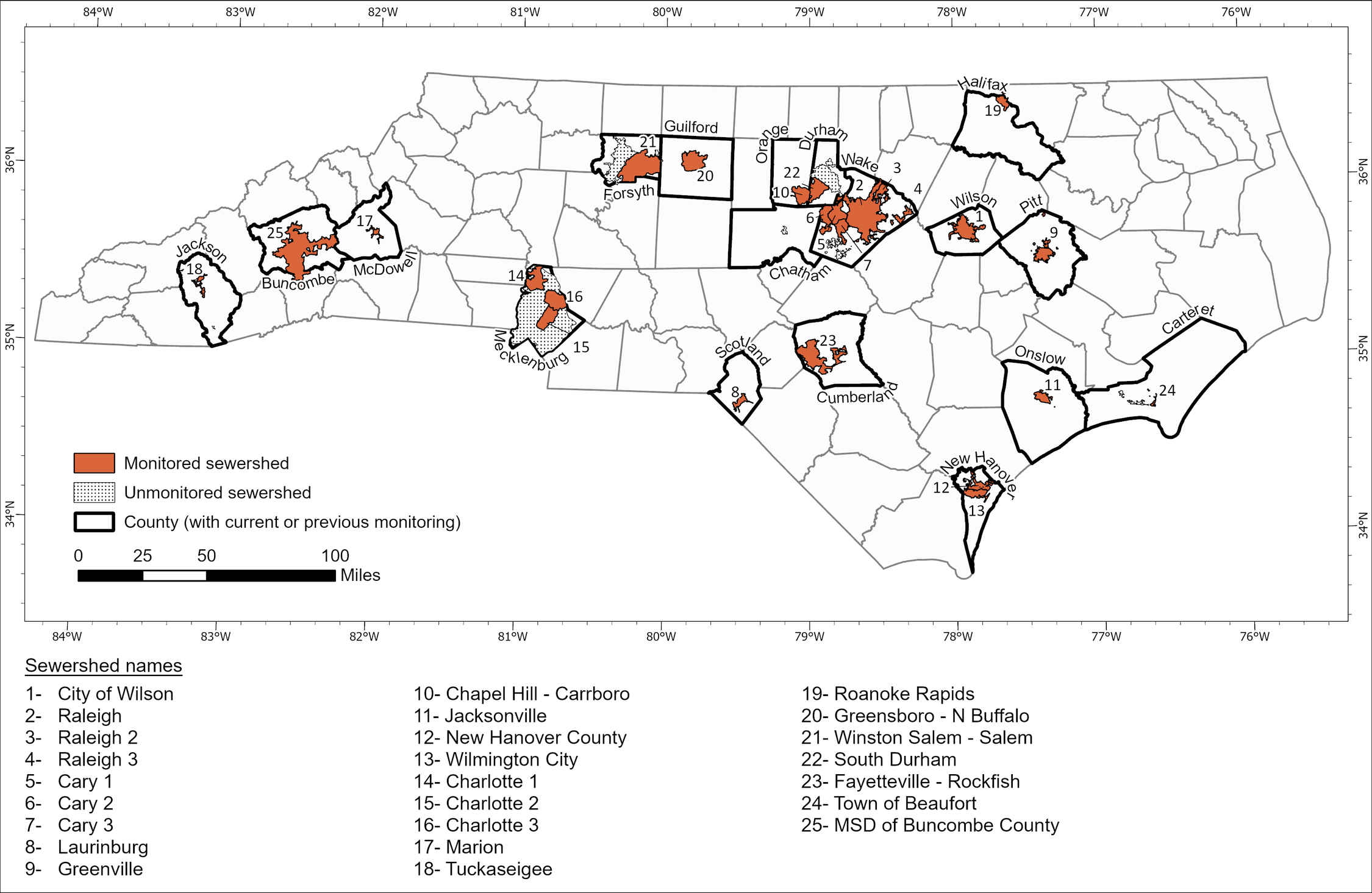

Supplement: S1 Fig — A sewershed boundary shows the area from which wastewater flows to a wastewater treatment plant sampling site. Monitored sewersheds were those participating in the North Carolina Wastewater Monitoring Network as of June 2022. Monitored sewersheds were combined with unmonitored sewersheds to create a single sewered area polygon for the county. North Carolina county boundaries are found at https://www.nconemap.gov/. (TIF) [file pone.0311516.s001.tif]
