# Supplementary material for: Equity in wastewater monitoring: Differences in the demographics and social vulnerability of sewered and unsewered populations across North Carolina
Source: PLoS One. 2024 Oct 10;19(10):e0311516. doi: 10.1371/journal.pone.0311516 (PMC11466389; doi:10.1371/journal.pone.0311516)
Supplement: Attachment — Submitted filename: ResponseToReviewers_PLOSOne_Recklingetal.docx [file pone.0311516.s007.docx]

Dear Editor and Reviewers,

Thank you for giving us the opportunity to submit a revised draft of the manuscript “Equity in wastewater monitoring: Differences in the demographics and social vulnerability of sewered and unsewered populations across North Carolina” for publication in the journal *PLOS One*. We appreciate the time and effort that you dedicated to providing feedback on our manuscript and are grateful for the insightful comments on and valuable improvements to our paper. We have incorporated most of the suggestions made by the reviewers. Please see below where we have addressed the comments point-by-point (our replies are in red) and correspondingly made changes to the manuscript.

**Editor’s Comments to the Author:**

Point 1: Please ensure that your manuscript meets PLOS ONE's style requirements, including those for file naming. The PLOS ONE style templates can be found at 
https://journals.plos.org/plosone/s/file?id=wjVg/PLOSOne_formatting_sample_main_body.pdf and 
https://journals.plos.org/plosone/s/file?id=ba62/PLOSOne_formatting_sample_title_authors_affiliations.pdf

**Author response:** Thank you for bringing this to our attention. We used the style templates linked above to make any necessary changes in the revised manuscript to match PLOS ONE’s style requirements. We un-bolded the title and indented each paragraph.

Point 2: Please provide an amended statement that declares *all* the funding or sources of support (whether external or internal to your organization) received during this study, as detailed online in our guide for authors at http://journals.plos.org/plosone/s/submit-now.  Please also include the statement “There was no additional external funding received for this study.” in your updated Funding Statement. 
Please include your amended Funding Statement within your cover letter. We will change the online submission form on your behalf.

**Author response:** We have included an amended funding statement in the cover letter. The funding statement should read as follows: “Financial support for this research was provided by the authors’ institutions—Mathematica, North Carolina Department of Health and Human Services, and North Carolina State University. Additionally, we gratefully acknowledge the funding support for the NC Wastewater Monitoring Network provided by the Centers for Disease Control and Prevention (CDC) National Wastewater Surveillance System (NWSS) through the Epidemiology and Laboratory Capacity (ELC) Cooperative Agreement. The funders had no role in study design, data collection and analysis, decision to publish, or preparation of the manuscript. There was no additional external funding received for this study.”

Point 3: We note that Figures 3 and S1 in your submission contain map images which may be copyrighted. All PLOS content is published under the Creative Commons Attribution License (CC BY 4.0), which means that the manuscript, images, and Supporting Information files will be freely available online, and any third party is permitted to access, download, copy, distribute, and use these materials in any way, even commercially, with proper attribution. For these reasons, we cannot publish previously copyrighted maps or satellite images created using proprietary data, such as Google software (Google Maps, Street View, and Earth). For more information, see our copyright guidelines: http://journals.plos.org/plosone/s/licenses-and-copyright.
 
We require you to either (1) present written permission from the copyright holder to publish these figures specifically under the CC BY 4.0 license, or (2) remove the figures from your submission: 
a. You may seek permission from the original copyright holder of Figures 3 and S1 to publish the content specifically under the CC BY 4.0 license.  
 
We recommend that you contact the original copyright holder with the Content Permission Form (http://journals.plos.org/plosone/s/file?id=7c09/content-permission-form.pdf) and the following text:
“I request permission for the open-access journal PLOS ONE to publish XXX under the Creative Commons Attribution License (CCAL) CC BY 4.0 (http://creativecommons.org/licenses/by/4.0/). Please be aware that this license allows unrestricted use and distribution, even commercially, by third parties. Please reply and provide explicit written permission to publish XXX under a CC BY license and complete the attached form.”
 
Please upload the completed Content Permission Form or other proof of granted permissions as an ""Other"" file with your submission.
 
In the figure caption of the copyrighted figure, please include the following text: “Reprinted from [ref] under a CC BY license, with permission from [name of publisher], original copyright [original copyright year].”
 
b. If you are unable to obtain permission from the original copyright holder to publish these figures under the CC BY 4.0 license or if the copyright holder’s requirements are incompatible with the CC BY 4.0 license, please either i) remove the figure or ii) supply a replacement figure that complies with the CC BY 4.0 license. Please check copyright information on all replacement figures and update the figure caption with source information. If applicable, please specify in the figure caption text when a figure is similar but not identical to the original image and is therefore for illustrative purposes only.

**Author response:** Thank you for pointing this out. The map images in Figures 3 and S1 do not use copyrighted material. The base maps in Figures 3 and S1 show North Carolina county boundaries, which are publicly available at <https://www.nconemap.gov/>. We have added the following language to the captions for Figures 3 and S1.

*“North Carolina county boundaries can be downloaded from* [*https://www.nconemap.gov/*](https://www.nconemap.gov/)*.”*

**Reviewer’s Comments to the Authors:**

**Reviewer 1**

Introduction section can be revised and include a discussion addressing the following points:
• Point 1: What were the main challenges to accessing clinical testing during the early COVID-19 pandemic, and how did these challenges alig-n with existing structural disparities?)

**Author response:** As suggested by the reviewer, we have added additional language about access to clinical testing and its alignment with structural disparities to the first paragraph of the Introduction as follows:

*“Access to testing—a critical public health resource—was aligned with known structural disparities, with fewer testing sites per person in neighborhoods with more Black, Latinx, and low-income residents, and inequities among minority, uninsured, and rural groups [1,2]. In poorer areas, testing sites were located farther away, [3] and in communities that were majority Black and Hispanic, residents were more likely to face longer wait times and understaffed testing centers, which limited their inclusion in early COVID-19 public health surveillance.”*

• Point 2: What are the potential limitations and equity concerns associated with wastewater monitoring, particularly in low- and middle-income countries (LMICs)?

**Author response:** We agree that this is an important point. In the third paragraph of the introduction (lines 55- 67, we discuss how wastewater monitoring is more limited in LMICs than high income countries (HICs) due to resource and infrastructure constraints. We added additional information about this topic with a relevant citation in lines 64-67.

*“In LMICs, wastewater monitoring is also less likely to be representative of the entire community because sampling is more commonly grab samples collected from surface waters, open drains, or pit latrines (versus composite samples collected from municipal wastewater treatment plants in HICs)* *[13].”*

Methodology Section:
• Point 3: In what ways could the methodology used in this study be improved or expanded to provide a more comprehensive understanding of the representativeness and equity of wastewater monitoring across different regions?

**Author response:** We appreciate the suggestion to think about ways to improve/expand our methodology to provide a more comprehensive understanding of the equity of wastewater monitoring in other regions. In lieu of including this information in the Methods sections, we added the following text in lines 356-361 in the new Future perspectives section:

*“The geospatial methods described in this study could be readily adapted to other states if a national sewershed database were developed, perhaps by building on the updated Clean Watershed Needs Survey conducted by the EPA [36]. This study’s methodology could also be expanded to include additional US Census variables or other indices, such as the area deprivation index [37], social deprivation index [38], and structural racism effect index [39], which are relevant to public health in that state or region.”*

In the results and discussion section, the following points can be explained with relevant citations:
• Point 4: How reliable is wastewater monitoring as a public health tool in representing the health markers and disease burden of an entire community, especially given the variations in sewershed populations?

**Author response:** Thank you for raising this question. We have added text to the discussion about how our results largely support the role of wastewater monitoring as a public health tool to understand disease burden of a community in lines 274-278:

*“Further, the strong correlation between county level and sewershed level COVID-19 clinical data points to the reliability of wastewater monitoring as a public health tool* *[14,23]. While studies on sewershed population fluctuations are still needed, a simulation study suggests that wastewater data from sewered communities can be indicative of health trends in neighboring unsewered areas [24].”*

• Point 5: What are the implications of finding that sewered populations are more vulnerable than unsewered populations on the overall effectiveness of wastewater monitoring in reflecting the true state of public health?

**Author response:** Thank you for bringing up this important point. We have reorganized the second paragraph in the discussion section to highlight the implications of this finding. We changed the text in lines 310-314 to the following:

*“Whether the disproportionate representation of vulnerable populations in wastewater monitoring is desirable depends on how the data will be used, but the potential overrepresentation is important to recognize when interpreting and communicating insights from wastewater data. Furthermore, given these disparities, wastewater monitoring data should be interpreted alongside other surveillance data to gain a more complete picture of the ‘true’ state of public health.”*

• Point 6: How do the demographic and social vulnerability differences between sewered and unsewered populations affect the accuracy and equity of health data derived from wastewater monitoring?

**Author response:** Thank you. We feel that this point has been addressed in our response to Point 5.

• Point 7: What measures can be taken to ensure that wastewater monitoring includes a representative sample of the entire population, especially in areas with significant differences between sewered and unsewered residents?

**Author response:** Thank you for recommending that we further address this topic. In the discussion section, we recommend that wastewater programs reassess the monitored sewershed population representativeness as sample site composition changes (lines 278-284) or surveillance goals are adapted over time (lines 373-384). We also added text in lines 370-372 of the Future perspectives section to describe how adding sentinel sites can improve representativeness in areas which lack sewer connectivity.

*“When disease hot spots and sewer connectivity do not overlap geographically, a sampling approach combining monitoring at centralized treatment plants with sampling at sentinel locations (such as schools and offices) [41] could improve the representativeness of wastewater data.”*

A section of future perspective can be included in the manuscript and following points can be highlighted:

- What are the potential limitations of relying on wastewater monitoring for public health surveillance, and how can these limitations be mitigated to improve the accuracy and inclusivity of the data?
- How might the differences between individual sewershed and county populations impact the interpretation of wastewater monitoring data for public health decision-making at the county and state levels?
- How can the findings of this study inform the development of policies and practices to enhance the use of wastewater monitoring as a tool for equitable public health surveillance and intervention?
- What role do socio-economic and infrastructural factors play in the observed differences between sewered and unsewered populations, and how can these factors be accounted for in future wastewater monitoring studies?

**Author response:** We think this is a great suggestion. We added a Future perspectives section at the end of the discussion section. The first paragraph of that section addresses several points raised by the reviewer (line 351-372).

*“As wastewater monitoring expands in geographic reach and utility, it will become increasingly important to describe in detail the populations residing in sewered areas**. Given previous research showing that the relationship between income and the use of decentralized wastewater systems varies across states [35], state wastewater programs should evaluate the characteristics of sewered and unsewered populations and carefully consider the implications of any differences to ensure that wastewater monitoring is executed equitably within the state.* *The geospatial methods described in this study could be readily adapted to other states if a national sewershed database were developed, perhaps by building on the updated Clean Watershed Needs Survey conducted by the EPA [36]. This study’s methodology could also be expanded to include additional US Census variables or other indices, such as the area deprivation index [37], social deprivation index [38], and structural racism effect index [39], which are relevant to public health in that state or region.* *Recognizing that sewershed populations are dynamic, wastewater monitoring programs should factor in known fluctuations in the size of the sewershed population due to seasonal tourism or major events, and broader chances in demographics and population mobility [24] to ensure that public health interventions are appropriately targeted. Although we found that areas with greater sewer connectivity have lower income, in some states, the opposite is true and sewer connectivity decreases with decreasing income [40]. Accordingly, when expanding wastewater monitoring to new sampling locations, officials should consider the role of structural inequalities and environmental justice [40]. One potential approach to enhancing the equity of wastewater monitoring for public health would be to consider whether sampling occurs in areas of high COVID-19 disease burden.* *When disease hot spots and sewer connectivity do not overlap geographically, a sampling approach combining monitoring at centralized treatment plants with sampling at sentinel locations (such as schools and offices) [41] could improve the representativeness of wastewater data.”*

**Reviewer 2**

Reviewer #2: This manuscript looks at differences in the demographics of sewered and unsewered populations across North Carolina. From the wastewater-surveillance and wastewater-based epidemiology aspect, I'm really unsure what the purpose is because of course the samples only represent those who contributed to them. There are other publications that have used census data and wastewater analysis to identify correlates of demographics which may have otherwise remained unknown which is what I was hoping this study would contribute to - but these seem to have been overlooked. As such I don't really see how this adds valuable information to the literature - but it is something that obviously should be considered when interpreting data in. It is written well though and the methodology used to determine catchment demographics is correct. BUT it also ignores that catchments are dynamic and that changes occur both in terms of short term and longer term - which is also important consideration for monitoring.

**Author response:** We want to thank this reviewer for their valuable feedback. We have addressed their concerns in several areas of the manuscript and hope the revision has enhanced the paper.

Line 80-84. To highlight the contribution of this study to the literature, we added the following text:

*“Existing investigations have been hindered by the lack of comprehensive sewershed geospatial data to define the community areas upstream of wastewater sampling sites. We contribute to the literature by leveraging the sewershed polygon data collected by the North Carolina Wastewater Monitoring Network (NCWMN) to conduct an empirical analysis comparing the sewered and unsewered populations.”*

Line 89-98. To clarify the main objective of the study, we added the following text to the last paragraph of the introduction section:

*“This study explores the demographic differences between sewered and unsewered populations in North Carolina, one of the first eight states funded by CDC NWSS, and any implications related to the representativeness and equity of wastewater monitoring for public health surveillance.” … “By highlighting the similarities and differences, we aim to improve the use of wastewater data for equitable public health action”*

Line 361-364. We recognize the limitation of not being able to consider the dynamic nature of wastewater catchment populations in our analysis, so we added the following text in the future perspective section.

*“Recognizing that sewershed populations are dynamic, wastewater monitoring programs should factor in known fluctuations in the size of the sewershed population due to seasonal tourism or major events, and broader changes in demographics and population mobility [24] to ensure that public health interventions are appropriately targeted.”*
