# Supplementary material for: Equity in wastewater monitoring: Differences in the demographics and social vulnerability of sewered and unsewered populations across North Carolina
Source: PLoS One. 2024 Oct 10;19(10):e0311516. doi: 10.1371/journal.pone.0311516 (PMC11466389; doi:10.1371/journal.pone.0311516)
Supplement: Attachment — Submitted filename: ResponseToReviewers2_Reckling.docx [file pone.0311516.s008.docx]

Dear Editor and Reviewers,

Thank you for allowing us to submit a revised draft of the manuscript “Equity in wastewater monitoring: Differences in the demographics and social vulnerability of sewered and unsewered populations across North Carolina” for publication in the journal *PLOS One*. We appreciate the time and effort you dedicated to providing feedback on our manuscript and are grateful for the insightful comments and valuable improvements to our paper. We have incorporated most of the suggestions made by the reviewers. Please see below where we have addressed the comments point-by-point (our replies are in red) and made changes to the manuscript.

**Editor’s Comments to the Author:**

Point 1: Journal Requirements:

Please review your reference list to ensure that it is complete and correct. If you have cited papers that have been retracted, please include the rationale for doing so in the manuscript text, or remove these references and replace them with relevant current references. Any changes to the reference list should be mentioned in the rebuttal letter that accompanies your revised manuscript. If you need to cite a retracted article, indicate the article’s retracted status in the References list and also include a citation and full reference for the retraction notice.

**Author response:** Thank you for bringing this to our attention. We checked that our references are published and accessible via the DOIs and URLs. We updated the following references’ URLs:

*Olesen SW, Young C, Duvallet C. The Effect of Septic Systems on Wastewater-Based Epidemiology. 2022 Oct. Available: https://biobot.io/publications/the-effect-of-septic-systems-on-wastewater-based-epidemiology/*

*CDC. Cases, Data, and Surveillance. In: Centers for Disease Control and Prevention [Internet]. 2023 [cited 26 Apr 2023]. Available:* *https://archive.cdc.gov/#/details?url=https://www.cdc.gov/coronavirus/2019-ncov/covid-data/investigations-discovery/hospitalization-death-by-race-ethnicity.html*

**Reviewer’s Comments to the Authors:**

**Reviewer #1: (No Response)**

We are glad we have addressed Reviewer #1’s previous comments. We want to thank them for the helpful feedback.

**Reviewer #3:**

• Point 1: One of the outcome of the study is that the sewered population is vulnerable than unsewered population. Is that because for the unsewered population, water quality is not monitored? Is it monitored? How is it monitored? Can that add to the data gap?

**Author response:** We appreciate this comment but would like to clarify that our paper focuses on comparing the demographics and social vulnerability of sewered and unsewered populations. Water quality was not included in the scope of this study.

• Point 2: In the case of COVID-19, even at the household level, if not all the members were infected, then how can wastewater monitoring be helpful than clinical assessment, which would provide individual-level assessment to take precautions? Although wastewater monitoring would be helpful in identifying county or state-level severity of the spread of disease; at the local or individual level, clinical assessment would be important. Then what is the contribution of this study? For whom the information obtained through this assessment would be beneficial?

**Author response:** Thank you for your suggestion. We agree with the reviewer that wastewater monitoring will not provide individual-level assessment but rather serves as a complementary approach to understanding community-level spread of the disease. In lines 46-54 of the Introduction, we discuss several benefits of wastewater monitoring compared to clinical testing. We added to the second paragraph of the Future Perspectives (lines 388-393) to highlight how officials can use information about the monitored sewered population to increase the utility of wastewater monitoring.

*“A better understanding of the characteristics of populations included in wastewater monitoring will also help officials use wastewater data effectively and adapt sampling strategies to address ongoing public health needs. Insights into the demographics and vulnerability of wastewater-monitored populations can enable tailored communications and interventions and equitable resource distribution. Furthermore, wastewater programs can use population information to effectively monitor additional pathogens such as influenza, respiratory syncytial virus, and monkeypox virus.”*

• Point 3: The results from the study can be briefly justified based on the authors’ experience or understanding

a. For example-

Line 260-263 - Educational attainment was lower in the sewered population than the unsewered population in seven counties (all but Forsyth County and Pitt County), ranging from a -7.0 to -0.2 pp difference. This difference was also statistically significant for Durham and Chatham counties (Table S4).

What is the reason for this? How is this expression related to the aim of the study? The authors can explain the results after stating them with the reason as per their experience or understanding.

**Author response:** We appreciate this feedback. We added a brief explanation of the results based on our knowledge of North Carolina’s wastewater program and two new references in lines 189-195

*“The observed differences may be related to how sites were enrolled in North Carolina’s wastewater monitoring program. The initial group of sites participating in the NCWMN came from a COVID-19 wastewater surveillance pilot project coordinated by universities [22], and so were located in urban centers near universities with labs that had the capacity to analyze wastewater samples. Over time, the NCWMN expanded to include sites in other areas of the state, including the rural mountainous region in Western North Carolina, and underserved communities with higher social vulnerability, low COVID-19 vaccination rates, or both [23].”*

• Point 4: The discussion section explains more about the result, limitations and future work; however, a generalized discussion about integrating the findings of the study to provide a comprehensive picture and its importance in the context of existing literature needs to be added apart from just one the statement in line number 319-320 “Furthermore, given these disparities, wastewater monitoring data should be interpreted alongside other surveillance data to gain a more complete picture of the ‘true’ state of public health”

**Author response:** We thank the reviewer for suggesting ways to improve the manuscript’s relevance. We added to the discussion section to include how this study fits in the context of existing literature in lines 273-280 and added one reference.

*“Wastewater data can be used to track disease trends in communities connected to a sewer system. However, research describing how sewered and unsewered populations differ is limited. One study utilized WWTP sewershed polygon data to examine differences in population sizes, but did not look at social vulnerability or demographic differences [24]. Another explored demographic and economic characteristics of US households connected to sewer aggregated to various Census geographies, but did not utilize sewershed boundary data [25]. The present study is the first to combine sewershed polygon data and several population-based spatial datasets to characterize sewered and unsewered populations and assess whether populations monitored by a state’s wastewater program represent broader populations.”*

• Point 5: Conclusion, is a stand-alone section that should also include the limitation and future scope. In the conclusion section, the authors have precisely stated the outcomes of the study. However, the authors also need to summarize the limitations and future scope to make it a distinct section for effective readership.

**Author response:** Thank you for bringing this to our attention. We re-worded the Conclusion to include limitations and future perspectives in lines 406-410.

*“The in-depth geospatial analyses described here provide a framework for evaluating the characteristics and representativeness of wastewater-monitored populations and can be adapted as additional geospatial data describing sewered areas and population characteristics become available. Understanding sewered population characteristics will help officials use wastewater data effectively for public health decision-making and adapt wastewater testing strategies to monitor for future pathogens.”*

• Point 6: Minor corrections:

a. At some places % or pp is written, be consistent about its use throughout the manuscript

**Author response:** Thank you. Lines 151-154 stated that % and pp have different meanings. We used percentage point (pp) when we calculated the difference for categorical variables and we used percent (%) for calculating the relative difference in continuous variables (e.g., median household income). We also confirmed that the correct abbreviations were used for variables throughout the paper.

b. Supplementary sheet – Include the title, authors and affiliation at the top of the supplementary sheet so that the file doesn't get lost when a reader downloads it.

**Author response:**

Thank you. We have added the title, authors, and affiliations to the top of the supplementary sheets.
